# EGCG and nimodipine improve the symptoms of AD by inhibiting [Ca2+]i in hippocampal neurons of APP/PS1 transgenic mice

**DOI:** 10.1186/1750-1326-7-S1-S30

**Published:** 2012-02-07

**Authors:** Shuang Wang, Mingyan Liu, Zhenjie Zhang, Weifan Yao, Qiushi Tang, Minjie Wei

**Affiliations:** 1 Department of Pharmacology, School of Pharmaceutical Sciences, China Medical University, Shenyang, P. R. China

## Background

Recent studies have indicated that an alteration in intracellular calcium homeostasis could contribute to the development of Alzheimer’s disease (AD) . Previous studies supported that sustained changes in intracellular free calcium concentration ([Ca^2+^]_i_ ) homeostasis could provide the final common pathway for the neuropathological changes associated with AD. Calcium influx is play a key role in calcium overload. Calmodulin (CaM)/ Calmodulin-dependent protein kinase II /IV (/CaMK II /IV) is very important to regulate calcium homeostasis in the nervous system. (–)-Epigallocatechin-3-gallate (EGCG), which is classified the catechin family and is one of the major polyphenol constituents of green tea. Research has shown that EGCG could reduce [Ca^2+^]_i_, lessen mitochondrial damage, thereby prevent calcium overload and protect neurons. Transgenic mouse models have been created with mutations in genes related to AD. APP/PS1 double-transgenic mice, provide a valuable model for evaluating the pathogenesis of AD. Therefore, this study was designed to study the effect of EGCG and nimodipine on cognitive behavior, [Ca^2+^]_i_ ,CaM and CaMK II /IV in APP/PS1 double-transgenic mice.

## Methods

Ten C57 mice and thirty APP/PS1 mice were randomly divided into four groups (n=10 each group): control group (C57 BL/6J, WT group), APP/PS1 group, EGCG (6 mg/kg) -treated APP/PS1 group and nimodipine (20 mg/kg) -treated APP/PS1 group. The mice were lavaged once daily for 4 weeks. After finishing all treatments, animals were evaluated by behavioral testing (Morris Water Maze and passive avoidance test). The protein expression of β-Amyloid (1-40) in the hippocampus was measured by immunohistochemical staining. Laser scanning confocalmicroscopy (LSCM) was used to observe the changes of [Ca^2+^]_i_. The protein expression of CaM, CaMK II /IV in the hippocampus was measured by Western blot.

## Results

The results by Morris Water Maze showed EGCG and nimodipine could significantly improve the learning and memory impairment of APP/PS1 double-transgenic mice, the lower escape latency, shorter path length and faster improvement were found in the navigation training for spatial acquisition. EGCG and nimodipine could significantly decreased the latency and the error time to enter the dark compartment in the passive avoidance test. The effect of EGCG is significantly stronger than nimodipine. The results by immunohistochemical staining of β-Amyloid (1-40) showed EGCG and nimodipine significantly decreased the express of β-Amyloid (1-40) in the hippocampus of APP/PS1 double-transgenic mice. The effect of EGCG is significantly stronger than nimodipine. EGCG and nimodipine significantly reduced [Ca^2+^]_i_ in the hippocampal neurons. The effect of EGCG was weaker than nimodipine. The results by western blot analysis showed that EGCG and nimodipine significantly decreased the express of CaM and increased the express of CaMK II /IV in the hippocampus of APP/PS1 double-transgenic mice. The effect of nimodipine is stronger than EGCG.

## Conclusions

The effect of EGCG to improve the learning and memory impairment of APP/PS1 double-transgenic mice is stronger than nimodipine. However, the degree of EGCG to reduce [Ca^2+^]_i_ and increase the expression of CaMK II /IV is weaker than nimodipine. It is suggested that other mechanism of EGCG to amendment AD maybe exist.

